# Effects of Different Assistive Seats on Ability of Elderly in Sit-To-Stand and Back-To-Sit Movements

**DOI:** 10.3390/healthcare9040485

**Published:** 2021-04-20

**Authors:** Shu-Zon Lou, Jia-Yuan You, Yi-Chuan Tsai, Yu-Chi Chen

**Affiliations:** 1 Department of Occupational Therapy, Chung Shan Medical University, Taichung 40201, Taiwan; szlou@csmu.edu.tw (S.-Z.L.); s977034@gmail.com (Y.-C.T.); 2 Occupational Therapy Room, Chung Shan Medical University Hospital, Taichung 40201, Taiwan; 3 Department of Physical Therapy, I-Shou University, Kaohsiung 82445, Taiwan; you0902@isu.edu.tw; 4 Department of Biomedical Engineering, HungKuang University, Taichung 433304, Taiwan

**Keywords:** assistive lifting seat, elder adults, sit-to-stand, back-to-sit

## Abstract

The ability to perform sit-to-stand (STS) and back-to-sit (BTS) movements is important for the elderly to live independently and maintain a reasonable quality of life. Accordingly, this study investigated the STS and BTS motions of 28 healthy older adults (16 male and 12 female) under three different seat conditions, namely nonassisted, self-designed lifting seat, and UpLift seat. The biomechanical data were acquired using a three-dimensional (3D) motion analysis system and force plates, and were examined by one-way repeated-measures ANOVA to investigate the effects of the different seat conditions on the joint angle, joint moments, and movement duration time (α = 0.05). No significant difference was observed in the STS duration among the three test conditions. However, the BTS duration was significantly increased in the UpLift seat condition. Moreover, the peak flexion angle of the hip during STS motion was also significantly higher in the UpLift condition. For both motions (STS and BTS), the lifting seats significantly decreased the knee and hip joint moments, but significantly increased the ankle joint moment. Moreover, compared to the nonassistive seat, both assistive lifting seats required a greater ankle joint strength to complete the STS and BTS motions.

## 1. Introduction

The ability to perform sit-to-stand (STS) and back-to-sit (BTS) movements efficiently is essential for elders seeking to live an independent life. Standing up demands a higher muscle strength and joint angle of the lower limbs than walking or climbing stairs [1]. However, the STS transition is one of the most physically demanding activities in daily life, and requires not only lower limb strength, but also good dynamic equilibrium [2]. Older people are more likely to struggle to carry out standing-up or sitting-down tasks than younger people as a result of impaired balance due to reduced muscle strength, stroke, Parkinson’s disease, and so on. Furthermore, elder adults who are unable to stand up independently are at greater risk of falls [3,4], and frequently suffer a reduced quality of life [5,6,7,8,9].

Many studies have explored the time required to sit down or stand up under different ages and/or strategies. In general, the results have shown that: (1) young people stand and sit for less time than seniors, (2) the STS duration is shorter than the BTS duration for both younger and older people, and (3) the STS time increases with a reducing chair height [1,10,11,12,13]. However, these studies considered only healthy subjects. Hassani et al. [10] observed that older adults with impaired functional capacities showed both a shorter BTS duration and a reduced trunk angle than younger subjects. In addition, the older subjects adopted different strategies to perform BTS, e.g., dropping onto the chair, as a result (it was presumed) of motor-planning impairments. Manckoundia et al. [14] studied the kinematics of STS and BTS motions in healthy older adults and older adults with Alzheimer’s disease (AD), and found that the duration of both tasks was shorter in the AD group than in the healthy older group. Moreover, the kinematic analysis revealed that the AD subjects exhibited a lower trunk angle than the healthy subjects in both STS and BTS.

Hughes et al. [15] studied the role of the knee extensor strength in rising from a chair in the functionally impaired elderly compared to young healthy adults. The results suggested that strength is a limiting factor in determining the minimum chair height from which the functionally impaired elderly may rise. Cheng et al. [5] investigated various fall status predictors in the young, old nonfallers, and old fallers, and found that young individuals had significantly higher lower limb muscle strength than old nonfallers or old fallers. Moreover, among the old nonfallers and old fallers, the old fallers had a lower muscle power and required a longer time to leave their seat. Rutherford et al. [7] found that the hip and knee joint angles of the elderly were significantly greater than those of young adults while standing up. The elderly also showed greater activity of the lower extremity muscles. It was hence inferred that the elderly expend more mechanical effort than the young in performing standing-up motions.

Many assistive devices are available to help the elderly, or patients with poor lower limb muscle strength, perform stand-up or sit-down tasks. Jeyasurya et al. [16] compared the performance of unassisted and assisted STS rise motions in the elderly population with bar-, arm-, waist-, and seat-assistance, respectively. The results showed that seat- and waist-assistance not only provided greater stability in the standing process than bar-assistance, but also effectively reduced the knee joint effort. However, among all the assistance methods, the participants expressed a preference for the seat and bar assists. Seat-assistance devices are currently the most common means of helping the elderly perform STS and BTS motions. However, while many types of assistive devices are available on the market, it is unclear which particular device is most effective for older adults [16].

Many different mechanism designs for lifting seats have been proposed, including booster seats, spring-loaded flap seats, and ejector chairs. However, while some studies have reported that seating devices with mechanical assists reduce the mechanical demands of the STS task, others have argued that these designs interfere with balance and cause the patients to adopt abnormal motion mechanisms [7,17,18,19]. Rutherford et al. [7] investigated the mechanics of STS transfer when using a portable lifting-seat device and concluded that most mechanism factors were reduced during the STS process. For example, the peak hip joint moment decreased by around 35% when using the lifting-seat device compared to the nonassisted seat. In addition, a time delay was found between power stopping and the moment at which the participants actually stood up when using the power-lifting seat. However, besides the study of Rutherford et al. [7], the literature contains little information on the effects of the structural design of assistive lifting-seats on the instability or abnormal motion phenomena of the elderly in performing STS and BTS tasks. Furthermore, even though Rutherford et al. compared the mechanical effects of assisted and nonassisted transfer in the performance of STS motion, no such investigation was performed for BTS transfer [7].

From the findings above, it is clear that the biomechanical effects of lifting-seat designs are not yet fully understood; particularly as regards their effects on BTS transfer. Accordingly, this study conducted an experimental investigation into the biomechanical effects of a self-designed lifting seat and compared the results with those obtained for the nonassisted case and a commercial pneumatic lifting-seat designed for the general elderly. In performing the investigation, it is hypothesized that the joint angles and joint moments of the lower extremity in the self-designed lifting seat condition are similar to those in the commercial lifting-seat condition during both STS and BTS movements and are lower than those in the nonassisted case. The experimental results provide useful insights into the efficacy of different lifting-seat designs in improving the efficiency of STS and BTS transfer motions in the general elderly.

## 2. Materials and Methods

### 2.1. Participants

Twenty-eight healthy elderly people (16 male and 12 female) participated in the study. The participants had an average age of 69.5 ± 3.49 years, height of 160.05 ± 8.42 cm, weight of 63.24 ± 10 kg, and knee height of 39 ± 2.4 cm. The inclusion criteria were specified as no cognitive impairment and no history of musculoskeletal or neurological disorders that would affect their ability to rise from a chair (e.g., hip replacement, rheumatoid arthritis, stroke, Parkinson’s disease, and so on). The tests were terminated if the subjects developed progressive angina, significantly reduced systolic pressure, dizziness, paleness, cyanosis, nausea, or excessive blood pressure, or if they simply wished to stop. Prior to the experiments, the patients were asked to provide formal written consent. The study was approved by the Human Body Research Ethics Review Committee (IRB) of Chung Shan Medical University, Taiwan.

### 2.2. Seat Devices and Height-Adjustable Chair

**Figure 1:**
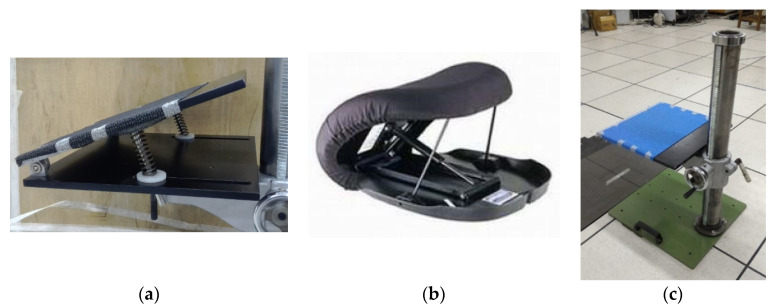
(**a**) Self-designed lifting seat with two 10 cm springs; (**b**) commercial lifting seat produced by Uplift Technologies, Inc. (Dartmouth, NS, Canada); (**c**) height-adjustable chair.

A self-designed lifting seat with two 10 cm springs was developed, as shown in Figure 1a. The springs had elastic coefficients of 0.158 kg/mm, and were fixed vertically beneath the seat with the help of two support rods. The tests were also performed using a commercial lift chair fitted with a hydro-pneumatic lift actuator (Uplift Technologies, Inc., Dartmouth, Nova Scotia, Canada) (Figure 1b). For both chairs, the tests were performed in a laboratory setting with a height-adjustable chair (Figure 1c).

### 2.3. Experimental Protocol

Before the subjects participated in the tests, their anthropometric data were acquired by an occupational therapist. The subjects were then familiarized with the experimental procedure and objectives, and introduced to the chair and assistive seats.

For each test, the three-dimensional (3D) lower extremity and trunk motions were recorded at 60 Hz using 6 cameras (Motion Analysis Corporation, Eagle Digital real-time system, Santa Rosa, CA, USA). In addition, the ground-reaction forces and moments were measured at 1200 Hz using two force plates (Bertec, BP 4550-08, Columbus, OH, USA) synchronized with the motion-analysis system. A total of 26 retroreflective spherical markers were attached to selected anatomic landmarks, including the spinous process of the 7th cervical vertebra, bilateral acromion process, bilateral epicondyle of the elbow joint, bilateral styloid process of the wrist joint, bilateral anterior superior iliac spine (ASIS), top of the sacrum, bilateral midthigh, bilateral lateral and medial femoral epicondyles, bilateral midshank, bilateral lateral and medial malleoli, bilateral heels, and bilateral toes between the second and third metatarsal heads.

The subjects were seated on the chair with the arms across the chest and the trunk erect. After placing each foot on one force plate, the chair height was adjusted to the shank positioned perpendicular to the ground. The subjects were instructed to stand up from the chair, maintain an upright position for 3 s, and then sit down again. Three seat conditions were tested, namely nonassisted (N), self-designed lifting (S), and UpLift seat (U). For each condition, at least three trials comprising the complete STS and BTS cycle were acquired for each subject.

### 2.4. Data Processing

Utilizing the data acquired from the experimental trials, 3D trajectories and the ground-reaction force were constructed using EVaRT 4.2 software (Motion Analysis Corporation, Santa Rosa, CA, USA). In addition, the joint angles; resultant forces; and moments of the ankle, knee, and hip joints in the sagittal plane were determined using self-developed kinematics and kinetics software in MATLAB (2018a, The Mathworks Inc., Natick, MA, USA). A four-segment model, including the foot, shank, thigh, and trunk, was employed in the analysis. In performing the analysis, each segment was assumed to be a rigid body, and the ankle, knee, and hip joints were treated as spherical joints. The joint angles were calculated based on the attached markers [20]. Meanwhile, the segment mass and inertia data were estimated by anthropometry [21]. In addition, the angular velocity and acceleration were calculated from the first and second derivatives of the joint angle, respectively [22]. The resultant loadings of the ankle, knee, and hip joints were then determined using an inverse dynamic procedure based on the Newton–Euler equations [20]. The joint angles and joint moments were smoothed using a low-pass digital filter at an estimated optimum cut-off frequency of 6.0 Hz. The approximate timing of the sit–stand–sit events was determined from an inspection of the ground-reaction-force data obtained from the force plates. The exact determinations of the start of standing up, seat-off, end of standing up, start of sitting down, seat-on, and end of sitting down were performed using the method described by Zijlstra [13]. All of the joint angles and moments calculated from the point of start of standing-up to the point of end of sitting-down were used for analysis purposes. The joint moments were normalized by the body weight times heights in every case (Nm/kg×m).

### 2.5. Data Analysis

One-way repeated-measures ANOVA analysis was performed to compare the complete time, kinematic, and kinetic parameters under the three different seat conditions. The difference between equivalent measurements was deemed to be significant if the corresponding *p* value was less than 0.05. A Bonferroni post hoc test was applied to determine where significant differences existed between the different conditions.

## 3. Results

### 3.1. Comparison of Phase Duration

#### 3.1.1. The Sit-to-Stand Duration

**Figure 2:**
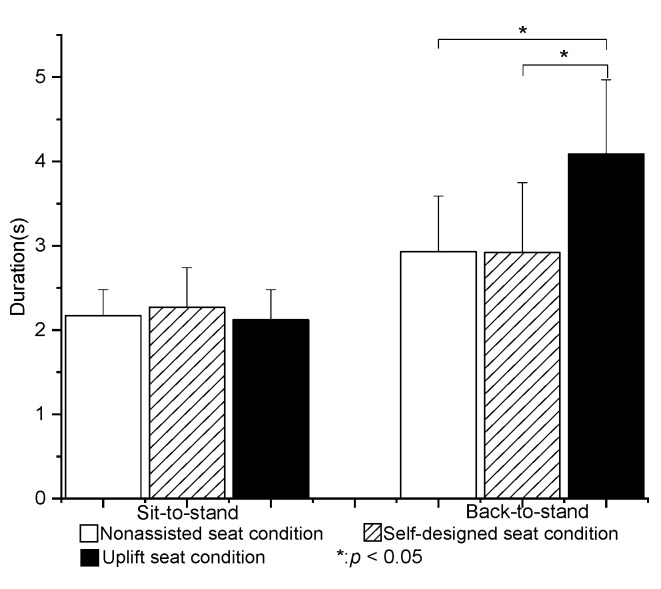
Means and standard deviations of sit-to-stand and back-to-sit durations for the three seat conditions. (* value is significantly difference).

The mean total STS duration in the nonassisted seat condition (N) was 2.17 ± 0.31 s. No significant difference was observed in the mean total STS duration among the three test conditions (*p* > 0.05) (Figure 2).

#### 3.1.2. The Back-to-Sit Duration

The mean total BTS duration in the N condition was 2.93 ± 0.66 s. The mean total BTS duration was significantly longer in the U condition than in the other two conditions (*p* < 0.05). However, no significant difference was found between the BTS durations of the N and S conditions (Figure 2).

### 3.2. Joint Angle

#### 3.2.1. STS Phase

**Figure 3:**
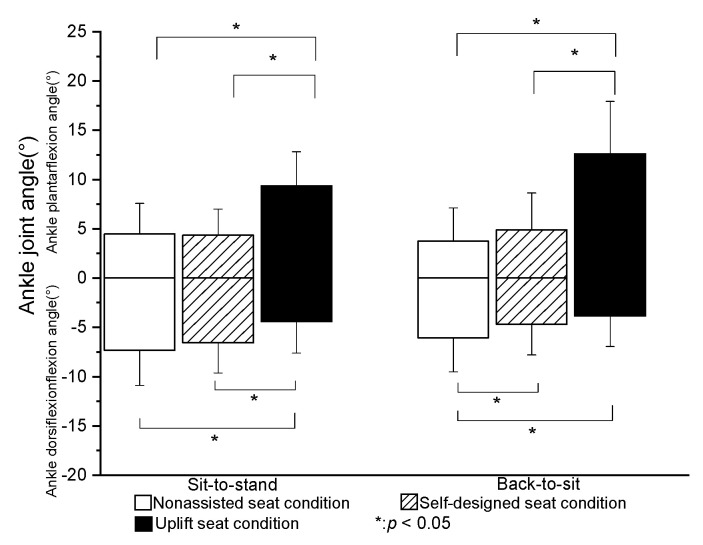
Means and standard deviations of peak ankle joint angle in STS and BTS tasks for the three seat conditions. (* value is significantly difference).

**Figure 4:**
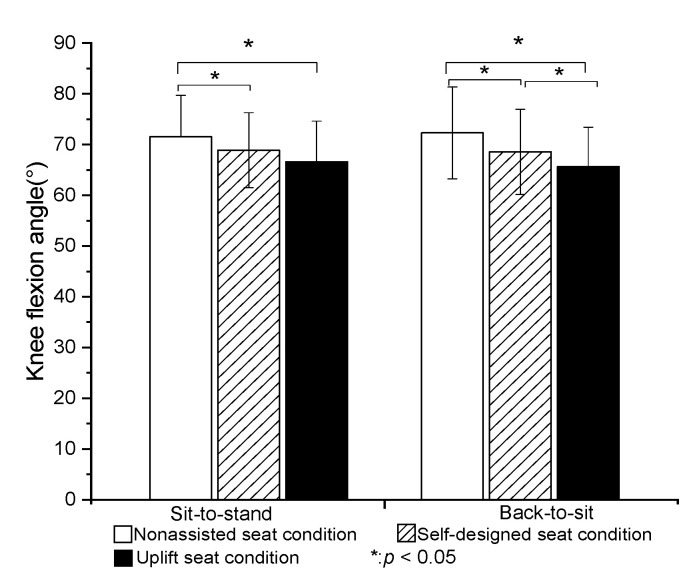
Means and standard deviations of peak knee flexion angle in STS and BTS tasks for the three seat conditions. (* value is significantly difference).

**Figure 5:**
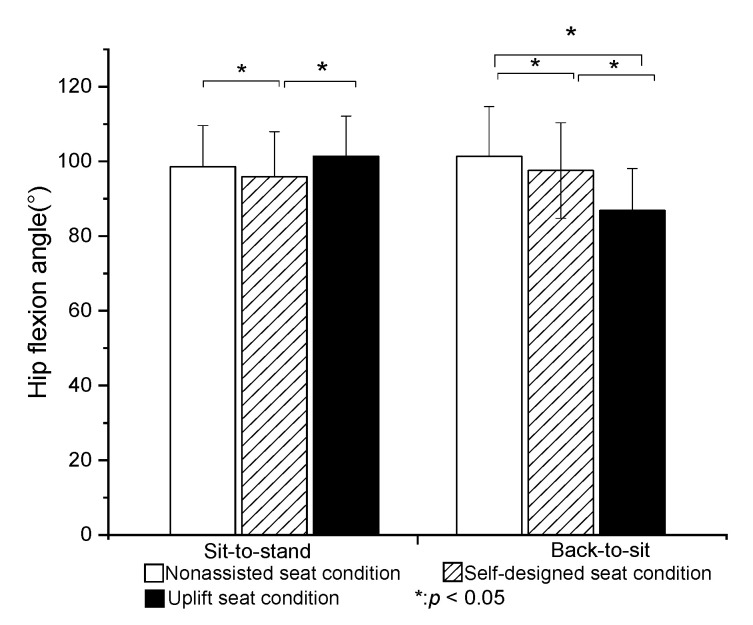
Means and standard deviations of peak hip flexion angle in STS and BTS tasks for the three seat conditions. (* value is significantly difference).

The peak plantar flexion angle of the ankle joint was significantly greater in the U condition than in the N or S condition (*p* < 0.05). However, no significant difference was found between the N and S conditions (Figure 3). The peak dorsiflexion angle of the ankle joint was significantly smaller in the U condition than in the N or S conditions (*p* < 0.05). The peak flexion angle of the knee was significantly lower in the S and U conditions than in the N condition (*p* < 0.05). However, no significant difference was found between the S and U conditions (Figure 4). Finally, the peak flexion angle of the hip was significantly higher in the U condition than in the S condition (*p* < 0.05). However, no significant difference was found between the N and U conditions (Figure 5).

#### 3.2.2. BTS Phase

The peak plantar flexion angle of the ankle was significantly higher in the U condition than in the N or S conditions (*p* < 0.05). However, no significant difference was found between the N and S conditions. The peak dorsiflexion angle of the ankle joint was significantly lower in the S and U conditions than in the N condition (*p* < 0.05). However, there was no significant difference between the S and U conditions (Figure 3). The peak flexion angles of the knee and hip joints obtained under the three test conditions were significantly different between each condition (*p* < 0.05) (Figure 4 and Figure 5).

### 3.3. Joint Moment

No significant differences were found between the left and right leg for the joint moments of the ankle, knee, and hip joints, respectively, whether standing up or sitting down (*p* > 0.05).

#### 3.3.1. STS Phase

The peak ankle joint moment was significantly higher under the S and U conditions than under the N condition (*p* < 0.05), whereas the peak hip joint moment was significantly lower (*p* < 0.05) (Table 1). However, no significant difference was observed between the S and U conditions (*p* = 0.38 and 0.37) for the peak ankle and hip joint moments. The peak knee joint moment was significantly different among the three seat conditions. In particular, the peak knee joint moment in the U condition was significantly lower than that in the S condition (*p* < 0.05). Similarly, the peak knee joint moment in the S condition was significantly lower than that in the N condition (*p* < 0.05).

#### 3.3.2. BTS Phase

The peak knee and hip joint moments in the S condition were significantly lower than those in the N condition (*p* < 0.05). The peak knee and hip joint moments in the U condition were significantly lower than those in the S condition (*p* < 0.05). The peak knee and hip joint moments in the S condition were also significantly lower than those in the N condition (*p* < 0.05). The peak ankle joint moment in the S condition was significantly higher than that in the N condition (*p* < 0.05). The peak ankle joint moment in the U condition was significantly higher than that in the S or N conditions (*p* < 0.05).

## 4. Discussions

The main purpose of this study was to compare the movement mechanics during STS and BTS transfer under different assistive seats.

### 4.1. Time of STS and BTS

The total STS time in the N condition (2.2 ± 0.3 s) was similar to that reported by Kuo et al., Millington et al., and Rutherford et al. [7,12,23]. Compared with the method used in the present study for defining each movement phase of the STS and BTS movements, Mourey et al. [11] determined the start and end points of the motion in accordance with a change of the exceeding a threshold of 10% of the peak forward angular velocity of the trunk. Thus, compared with the present study, the starting point was later, while the ending point was earlier. Consequently, the overall STS time (1.82± 0.31 s) was relatively shorter than that in the present study. The mean total STS durations for the three seat conditions in the present study were 2.17 ± 0.31 s (N), 2.27 ± 0.47 s (S), and 2.12 ± 0.36 s (U). No significant difference existed between them (*p* > 0.05). The results were thus consistent with the findings of Rutherford et al. [7] that assistive devices did not significantly affect the total STS movement time.

Few studies have investigated the BTS duration in healthy seniors. However, Mourey et al. [24] reported that the duration of sitting down in elderly subjects was around 1.69 ± 0.31 s. Zijlstra et al. [13] measured the sitting-down duration in older adults and patients with Parkinson’s disease using force plates and body-fixed sensors. The force-plate results showed that the BTS duration was around 1.93 ± 0.4 s. By contrast, the body sensor results indicated a slightly longer duration of approximately 2.09 ± 0.25 s. It was noted that both durations were lower than the BTS time measured in the present study in the N condition (2.86 ± 0.82 s). The inconsistency among these results most likely stemmed from different motion settings and/or data-processing methods in these studies. However, in these studies, the sitting-down time was longer than the standing-up time.

In the present study, the BTS duration increased significantly when using the UpLift seat. After completing the STS motion, the seat was in a fully expanded condition. Thus, to perform BTS motion, the subjects had to overcome the resistive force produced by the hydraulic mechanism of the UpLift seat. Accordingly, during BTS transfer, the subjects were required to shift their weight to a more rearward position of the seat cushion in order to apply their full body weight to the seat and drive it in a downward direction. The adjustment in the sit-down posture thus increased the BTS time compared to that in the nonassisted and self-designed lifting-seat conditions.

No significant difference was noted between the BTS times for the nonassisted and self-designed lifting seats. However, the self-designed seat provided better support than the nonassisted seat, and provided a faster BTS time than the UpLift seat. The faster BTS time implies that the subjects experienced less resistance in returning the seat to the seated position. Consequently, it was inferred that the self-designed lifting seat was better suited to the needs of the elderly than the commercial UpLift seat.

### 4.2. Joint Angle

The present results showed that the self-designed lifting seat significantly reduced the flexion angles of the hip and knee (by 3% and 2%, respectively) during STS transfer compared to the nonassisted seat. Compared to the self-designed lifting seat and nonassisted seat, the UpLift seat significantly reduced the knee flexion angle during STS motion, but significantly increased the hip flexion joint angle. The UpLift seat also significantly increased the ankle plantar flexion angle compared with the nonassisted seat and self-designed lifting seat. These findings differed from those of Rutherford et al. [7], who reported that the hip joint angle decreased significantly when using a Power Seat UpLift chair during STS, while the knee joint angle decreased by around 18% and the ankle joint dorsiflexion angle increased by 5%. In the present study, the knee joint angle reduced by only around 7% when using the UpLift seat compared to the N condition. The difference between the two studies for the hip flexion angle may be the result of the use of electric power in controlling the lifting seat. In particular, the participants were elevated passively by the lifting seat, and therefore did not need to shift their center of gravity to activate the hydro-pneumatic lift actuator in the early stage of STS transfer. Only once the power seat stopped raising were the subjects required to stand up. In other words, they stood up from a higher seat position in the later stage of STS. As a result, the hip flexion angle was less than that produced by the UpLift seat in the current study.

For the UpLift seat, the user must sit on the full seat to start the hydraulic actuator. However, while this strategy significantly reduces the knee joint angle and dorsi-flexion angle of the ankle, the need for the user to move the center of gravity of the body to complete the STS movement results in a higher hip flexion angle. By contrast, for the self-designed lifting seat used in the present study, the initial sitting posture of the participants was independent of the mechanical power developed by the seat. Consequently, the participants were observed to sit on only 1/3~2/3rd of the seat during STS, and to stand up with a smaller hip flexion angle as a result.

Overall, the present results indicated that the flexion angles of the knee and hip joints were significantly reduced in BTS transfer when using the self-designed lifting seat or UpLift seat. Moreover, the knee flexion angle when using the UpLift seat was significantly smaller than that when using the self-designed lifting seat. The latter finding can be attributed most probably to the difference in the unfolded heights of the two seats. In particular, the UpLift seat has a fully unfolded height of more than 25 cm, whereas the self-designed lifting seat has an unfolded height of just 20 cm. As a result, the users contacted the UpLift seat earlier in the BTS transfer process than the self-designed lifting seat, and therefore exhibited lower flexion angles of the knee and hip joints. It was noted that this tendency was similar to that reported in previous studies on the effects of higher chairs [9,23].

### 4.3. Joint Moment

Yoshioka et al. found that the peak ankle moment appeared in the late stage of STS movement [9], and was related primarily to a forward/backward tilting of the body movement rather than a vertical movement. As a result, the authors speculated that the main ankle joint moment played an important role in maintaining balance during the late stage of standing up. The results obtained in the present study showed that, for both STS and BTS movements, the ankle joint moment increased significantly when a lifting seat was used. In other words, the subject must apply a greater muscle strength of the ankle joint to maintain balance during STS and BTS transfer when using a lifting seat. However, the results also showed that both lifting seats significantly reduced the knee and hip joint moments compared to the nonassisted case. Thus, both seats reduced the required muscle strength of the knee and hip joints in performing STS or BTS transfer.

The self-designed seat and UpLift seat both significantly reduced the knee and hip joint moments during BTS transfer. However, the self-designed seat reduced the two moments by around 7% and 5%, respectively, while the UpLift seat reduced the moments by 21% and 15%, respectively. In other words, the UpLift seat provided a more effective reduction in the knee and hip joint moment during BTS than the self-designed seat. However, even though the UpLift seat significantly reduced the knee and hip joint moments, it increased the ankle joint moment by 39%. In other words, the UpLift seat required the application of greater ankle joint strength than the self-designed lifting seat when performing BTS movement.

Rutherford et al. reported that the Power Seat UpLift seat significantly reduced the maximum hip joint moment of both young and old subjects when standing up [7]. This finding is consistent with the present results, which showed that both the UpLift seat and the self-designed lifting seat significantly reduced the hip joint moment in STS transfer. However, compared with the electrically powered seat used in Rutherford’s study, which reduced the maximum hip joint moment by around 35%, the Uplift and self-designed lifting seats used in the present study reduced the maximum hip moment by just 9 and 11%, respectively. The inferior performance of the two seats may have arisen because both seats required the user to actively perform motion in order to actuate the hydraulic tube (UpLift chair) or springs (self-designed lifting seat) during STS. Furthermore, compared to the fully automatic, electrically powered lifting seat considered by Rutherford et al., the two seats used in the present study both require positive action on the part of the user to activate the assistive mechanism. Consequently, the lower reduction in the maximum hip joint moment observed in the present study may also reflect a lack of familiarity with the function and proper operation of the two seats.

### 4.4. Limitation

Sitting and standing-up tasks are both related to the strength of the lower limbs. However, in the recruitment process, the present study performed screening based only on the physiological history of the participants. That is, no account was taken of the subjects’ balance abilities and muscle strength, or their exercise habits. Intuitively, individuals with an exercise habit or greater muscle strength of the lower limbs may be expected to perform better in more challenging situations, such as low seat heights or the absence of assistive devices, than subjects with no exercise habits or reduced muscle strength. Accordingly, future studies may usefully analyze and compare the STS and BTS performance of participants for different seat heights and muscle strengths.

In practice, lifting-seat devices can be used not only by the elderly in normal home or institution environments, but also as a training device for the disabled, such as stroke patients. In other words, they can be used as a tool for training users with weak lower limb muscles to stand up or sit down on their own with the aid of assistive devices. However, the present study considered only healthy elderly people. Therefore, further studies should also examine the biomechanical effects of assisted seats on individuals with disabilities.

## 5. Conclusions

This study investigated the biomechanical effects of three seat conditions (nonassisted, self-designed lifting seat, and UpLift seat) on the performance of STS and BTS motions by 28 healthy older adults. The results showed that the self-designed lifting seat had no significant effect on the STS and BTS duration compared to the unassisted seat, but significantly reduced the flexion angle and joint moment of the knee and hip joints. However, the reduction in the moment of the knee and hip joints during BTS was less significant than that induced by the UpLift seat.

The UpLift seat significantly reduced the knee and hip moments compared to the unassisted condition. However, the user must sit on the full seat in order to activate the actuator and commence the lifting motion. In other words, the user is required to shift their center of gravity in order to complete the STS movement, and this leads to a greater hip flexion angle. Furthermore, the need to adjust the sit-down posture also increases the duration of the BTS movement.

Although both lifting seats significantly reduced the knee and hip joint moments, they also increased the ankle joint moment in BTS movement. The increase in the ankle joint moment was particularly significant when using the UpLift seat. Hence, it was inferred that the UpLift seat required more ankle joint strength than the self-designed lifting seat to actively balance the BTS movement.

## Figures and Tables

**Table 1:** Peak joint moment values in STS and BTS tasks for the three seat conditions.

Peak Joint Moment (Nm/kg×m) (Mean ± SD)						
Sit-To-Stand				Back-To-Sit		
Seats	Ankle	Knee	Hip	Ankle	Knee	Hip
N	0.269 ± 0.088	0.331 ± 0.106	0.613 ± 0.125	0.175 ± 0.075	0.350 ± 0.088	0.569 ± 0.125
S	* 0.288 ± 0.075	* 0.294 ± 0.010	* 0.544 ± 0.138	* 0.188 ± 0.063	* 0.325 ± 0.094	* 0.538 ± 0.131
U	* 0.288 ± 0.069	^§,^* 0.213 ± 0.082	* 0.556 ± 0.163	^§,^*0.244 ± 0.050	^§,^* 0.275 ± 0.069	^§,^* 0.481 ± 0.106

N: nonassistive condition; S: self-designed lifting-seat condition; U: UpLift seat condition. *: Significant difference compared to N. ^§^: Significant difference between S and U. SD: Standard deviation.

## Data Availability

The data presented in this study are available on request from the corresponding author. The data are not publicly available due to privacy reasons.
